# Association between hydrocarbon exposure and risk of stroke: a systematic literature review

**DOI:** 10.1186/s12883-025-04083-x

**Published:** 2025-02-21

**Authors:** Muhammed Shabil, Abhay M. Gaidhane, Nasir Vadia, Soumya V. Menon, Kattela Chennakesavulu, Rajashree Panigrahi, Ganesh Bushi, Diptismita Jena, Anju Rani, Sanjit Sah, Mahendra Singh, Prakasini Satapathy, Khang Wen Goh, Nosaibah Razaqi

**Affiliations:** 1 University Center for Research and Development, Chandigarh University, Mohali, Punjab India; 2 Faculty of Data Science and Information Technology, INTI International University, Nilai, Malaysia; 3 Jawaharlal Nehru Medical College, and Global Health Academy, School of Epidemiology and Public Health. Datta Meghe Institute of Higher Education, Wardha, India; 4 Department of Pharmaceutical Sciences, Faculty of Health Sciences, Marwadi University Research Center, Marwadi University, Rajkot, Gujarat 360003 India; 5 Department of Chemistry and Biochemistry, School of Sciences, JAIN (Deemed to be University), Bangalore, Karnataka India; 6 Department of Chemistry, Sathyabama Institute of Science and Technology, Chennai, Tamil Nadu India; 7 Department of Microbiology, IMS and SUM Hospital, Siksha ’O’ Anusandhan (Deemed to be University), Bhubaneswar, Odisha 751003 India; 8 Chitkara Centre for Research and Development, Chitkara University, Solan, Himachal Pradesh 174103 India; 9 School of Pharmaceutical Sciences, Lovely Professional University, Phagwara, India; 10 Centre of Research Impact and Outcome, Chitkara University, Rajpura, Punjab 140417 India; 11 Division of Research and Innovation, Uttaranchal University, Dehradun, India; 12 Department of Microbiology, Graphic Era (Deemed to be University), Clement Town, Dehradun, 248002 India; 13 Department of Paediatrics, Dr. D. Y. Patil Medical College Hospital and Research Centre, Dr. D. Y. Patil Vidyapeeth (Deemed-to-be-University), Pimpri, Pune, Maharashtra 411018 India; 14 Department of Public Health Dentistry, Dr. D. Y. Patil Medical College Hospital and Research Centre, Dr. D. Y. Patil Vidyapeeth (Deemed-to-be-University), Pimpri, Pune, Maharashtra 411018 India; 15 Department of Medicine, Korea Universtiy, Seoul, South Korea; 16 Center for Global Health Research, Saveetha Institute of Medical and Technical Sciences, Saveetha Medical College and Hospital, Saveetha University, Chennai, India; 17 Noida Institute of Engineering and Technology (Pharmacy Institute), Greater, Noida India; 18 Medical Laboratories Techniques Department, AL-Mustaqbal University, Hillah, Babil, 51001 Iraq; 19 Faculty of Data Science and Information Technology, INTI International University, Nilai, Malaysia; 20 Faculty of Mathematics and Natural Sciences, Universitas Negeri Padang, Padang, Indonesia; 21 Afghanistan Center for Epidemiological Studies, Herat, Afghanistan; 22 Faculty of Medicine, Ghalib University, Herat, Afghanistan; 23 Scientific Research Committee, Afghanistan Medical Students Association, Herat, Afghanistan

**Keywords:** Stroke, Hydrocarbon exposure, Polycyclic aromatic hydrocarbons, Volatile organic compounds, Environmental health

## Abstract

**Background:**

Hydrocarbon exposure, including polycyclic aromatic hydrocarbons (PAHs) and volatile organic compounds (VOCs), is increasingly linked to vascular dysfunction and stroke, a leading cause of morbidity and mortality globally. Common in occupational and environmental contexts, hydrocarbons induce oxidative stress, systemic inflammation, and endothelial dysfunction, disrupting vascular health. This systematic review examines the association between hydrocarbon exposure and stroke, emphasizing specific metabolites and their cerebrovascular effects.

**Methods:**

A comprehensive search across PubMed, Embase, and Web of Science was conducted through December 10 2024, identifying observational studies exploring hydrocarbon exposure and stroke risk. Studies meeting predefined inclusion criteria, excluding those with major methodological flaws, were synthesized narratively. Variations in hydrocarbon types, population demographics, and stroke outcomes were considered.

**Results:**

Six studies, including five cross-sectional and one retrospective cohort, with sample sizes ranging from 5,537 to 283,666 participants, demonstrated significant associations between hydrocarbon exposure and stroke risk. Key findings revealed strong associations for metabolites like 1-hydroxynaphthalene (OR: 1.89; 95% CI: 1.62–2.20) and 2-hydroxyfluorene (OR: 1.94; 95% CI: 1.66–2.26). However, variability in findings was noted, attributed to differences in study design, exposure levels, and populations studied.

**Conclusion:**

This review highlights a complex relationship between hydrocarbon exposure and stroke risk, with some studies indicating significant associations and others reporting inconsistencies. Standardized, large-scale research is essential to clarify this relationship, identify high-risk populations, and guide public health strategies to mitigate exposure and prevent stroke.

**Clinical trial number:**

Not applicable.

**Supplementary Information:**

The online version contains supplementary material available at 10.1186/s12883-025-04083-x.

## Introduction

Stroke affects over 15 million people annually and is a leading cause of disability and death worldwide [1, 2], accounting for approximately 6.5 million deaths each year. This condition, caused by interrupted blood flow to the brain, can manifest in different forms, including ischemic stroke, which represents about 87% of cases, hemorrhagic stroke, and transient ischemic attacks (TIAs) [3], often referred to as “mini-strokes.” Stroke imposes severe neurological, social, and economic burdens, with global costs exceeding $891 billion annually [4]. Ischemic stroke is a heterogeneous condition comprising distinct subtypes, including atherothrombotic infarct, cardioembolic stroke, lacunar infarct, infarct of unusual etiology, and essential cerebral infarct. These subtypes differ in their pathophysiology, prognosis, and response to treatment. However, most epidemiological studies do not differentiate between these subtypes when assessing environmental risk factors, making it challenging to determine whether certain stroke mechanisms are more vulnerable to hydrocarbon exposure [5]. While well-known risk factors such as hypertension, diabetes, smoking, and physical inactivity are extensively studied, emerging evidence highlights environmental exposures, such as air pollution and occupational hazards, as significant contributors. With nearly 70% of stroke cases occurring in low and middle-income countries, addressing these lesser-known triggers is essential for developing effective prevention strategies.

Hydrocarbons, organic compounds composed of hydrogen and carbon, are increasingly recognized in environmental health research for their potential effects on vascular and neurological systems [6]. These compounds, derived mainly from fossil fuel combustion, are pervasive in urban and industrial settings [6]. Hydrocarbons include several subcategories, with polycyclic aromatic hydrocarbons (PAHs) such as benzo[a]pyrene, naphthalene, phenanthrene, and fluoranthene and volatile organic compounds (VOCs) being of particular concern due to their abundance, reactivity, and toxicity [7]. Sources of hydrocarbon pollution range from vehicle emissions and industrial activities to residential heating and tobacco smoke [8]. Exposure occurs through various pathways, including inhalation, dermal absorption, and ingestion, making it an unavoidable aspect of modern life, particularly for individuals working in industries such as petrochemicals and construction.

Emerging research has revealed a concerning link between hydrocarbon exposure and an increased risk of stroke, highlighting the complex ways these compounds affect vascular health [9]. Hydrocarbons, particularly PAHs and VOCs, generate reactive oxygen species (ROS), leading to oxidative stress that damages blood vessels and disrupts vascular homeostasis [10]. This, combined with systemic inflammation triggered by hydrocarbons, exacerbates endothelial dysfunction, arterial stiffness, and atherosclerosis, all of which elevate the risk of thrombotic events [11, 12]. By interfering with key vascular pathways, such as nitric oxide signaling, hydrocarbons further impair circulation and increase susceptibility to stroke [13]. These interconnected mechanisms emphasize the need for further research to clarify this association and guide effective interventions.

Despite the growing body of research, the relationship between hydrocarbon exposure and stroke risk remains insufficiently understood, with studies yielding mixed findings. While some investigations indicate a strong association between hydrocarbon exposure and increased stroke incidence [14], others report minimal or non-significant correlations [15, 16]. These discrepancies likely stem from variations in study design, exposure measurement techniques, and the influence of confounding variables, such as co-exposure to other pollutants or individual susceptibility factors. These inconsistencies emphasizes the need for a systematic review to synthesize existing evidence, evaluate the strength of the association, and identify high-risk populations.

This systematic review aims to comprehensively analyze the relationship between hydrocarbon exposure and stroke risk, identifying vulnerable populations and underlying mechanisms. By synthesizing existing evidence, this review will enhance our understanding of the role of environmental factors in stroke risk and provide guidance for developing effective public health strategies to mitigate the associated burden.

## Methods

### Study design

This systematic review was designed to systematically evaluate and critically appraise the existing research on the association between hydrocarbon exposure and the risk of stroke. The review adhered to the framework outlined by the Preferred Reporting Items for Systematic Reviews and Meta-Analyses (PRISMA) [17] guidelines, ensuring a transparent and methodologically rigorous approach throughout the process (Table S1). Additionally, the review protocol was pre-registered with the International Prospective Register of Systematic Reviews (PROSPERO), providing a structured, standardized, and pre-specified approach to enhance the reliability and validity of the findings.

### Data sources and search strategy

A systematic search was performed across several electronic databases, including PubMed, Web of Science, and Embase, to locate studies examining the connection between hydrocarbon exposure and stroke risk. The search included all publications available up to December 10 2024. A combination of free-text terms and controlled vocabulary was utilized, with keywords such as (“polycyclic aromatic hydrocarbons” OR “PAHs” OR “polynuclear aromatic hydrocarbons” OR “aromatic hydrocarbons” OR “volatile organic compounds” OR “hydrocarbon exposure” OR “occupational exposure to hydrocarbons”) AND (“stroke” OR “ischemic stroke” OR “hemorrhagic stroke” OR “transient ischemic attack” OR “cerebrovascular disease”). Boolean operators (AND, OR) were applied strategically to ensure a thorough and systematic identification of relevant studies. No restrictions on language or publication type were applied to allow for an inclusive search process. Details of the full search strategy, including database-specific terms and adjustments, are provided in Table S2.

### Eligibility criteria

The selection process for studies was based on predefined inclusion and exclusion criteria. To be eligible for inclusion, studies had to meet the following criteria: [1] observational studies, such as cohort, case-control, or cross-sectional designs, that explored the relationship between hydrocarbon exposure (including PAHs, VOCs, or other forms of hydrocarbons) and stroke; [2] studies providing quantitative data, including odds ratios (OR), relative risks (RR), hazard ratios (HR), or sufficient information to calculate these metrics; [3] research that focused on both occupational and environmental exposure settings, such as petrochemical industries or air pollution; and [4] peer-reviewed articles with no restrictions on language or type of publication. Stroke was defined as including ischemic stroke, hemorrhagic stroke, and TIAs, following clinical or diagnostic criteria aligned with established guidelines. Studies excluded from this review were case reports, conference abstracts, opinion pieces, and review articles, to ensure that only primary research offering direct evidence of the hydrocarbon-stroke association was included.

### Study selection

Two researchers independently screened the titles and abstracts of all studies identified during the systematic search to evaluate their eligibility based on the established criteria. Full-text articles of studies considered potentially relevant were obtained and reviewed by the same two researchers to determine their final inclusion. Any disagreements that arose during the selection process were resolved through discussion, and when consensus could not be reached, a third researcher was consulted to provide a final decision.

To improve the accuracy and efficiency of the selection process, semi-automated software tools (e.g., Nested-Knowledge, MN, USA) were employed to remove duplicate records and facilitate the initial screening. This systematic approach ensured a thorough and reliable review of all studies investigating the link between hydrocarbon exposure and stroke risk.

### Data extraction and quality assessment

Data extraction was conducted independently by two authors utilizing a pre-developed standardized data extraction form. This form was tested on a subset of included studies to ensure its comprehensiveness and refined accordingly. The extracted data included general information such as study title, publication year, and first author, along with detailed study characteristics like design, setting, and sample size. Participant demographics, types of hydrocarbon exposure, exposure assessment methods, stroke outcomes, and covariates adjusted in the analysis were also meticulously recorded. Any discrepancies during the extraction process were resolved through discussion, or a third author was consulted to achieve consensus. Quality assessment of the included studies was independently using the Newcastle-Ottawa Scale (NOS) for non-randomized studies in meta-analyses. This scale evaluates studies across three dimensions: selection of participants, comparability of groups, and assessment of outcomes, with a maximum possible score of nine stars (Table S3).

### Data synthesis

The data synthesis for this systematic review was predominantly quantitative, aimed at summarizing effect estimates such as ORs, HRs, and other relevant metrics reported in the included studies. Due to differences in study designs, methods of exposure assessment, and outcome definitions, a meta-analysis was not feasible. Instead, the review focused on identifying consistent patterns and trends in the relationship between hydrocarbon exposure and stroke risk. The synthesis process involved analyzing and comparing ORs and HRs along with their confidence intervals (CIs) across studies to highlight discrepancies in effect estimates and investigate potential factors contributing to these variations. Key quantitative results, including ORs and HRs, were compiled into tables to facilitate direct comparisons and to emphasize the broader trends in the literature concerning the association between hydrocarbon exposure and stroke.

## Results

### Literature search

**Fig. 1:**
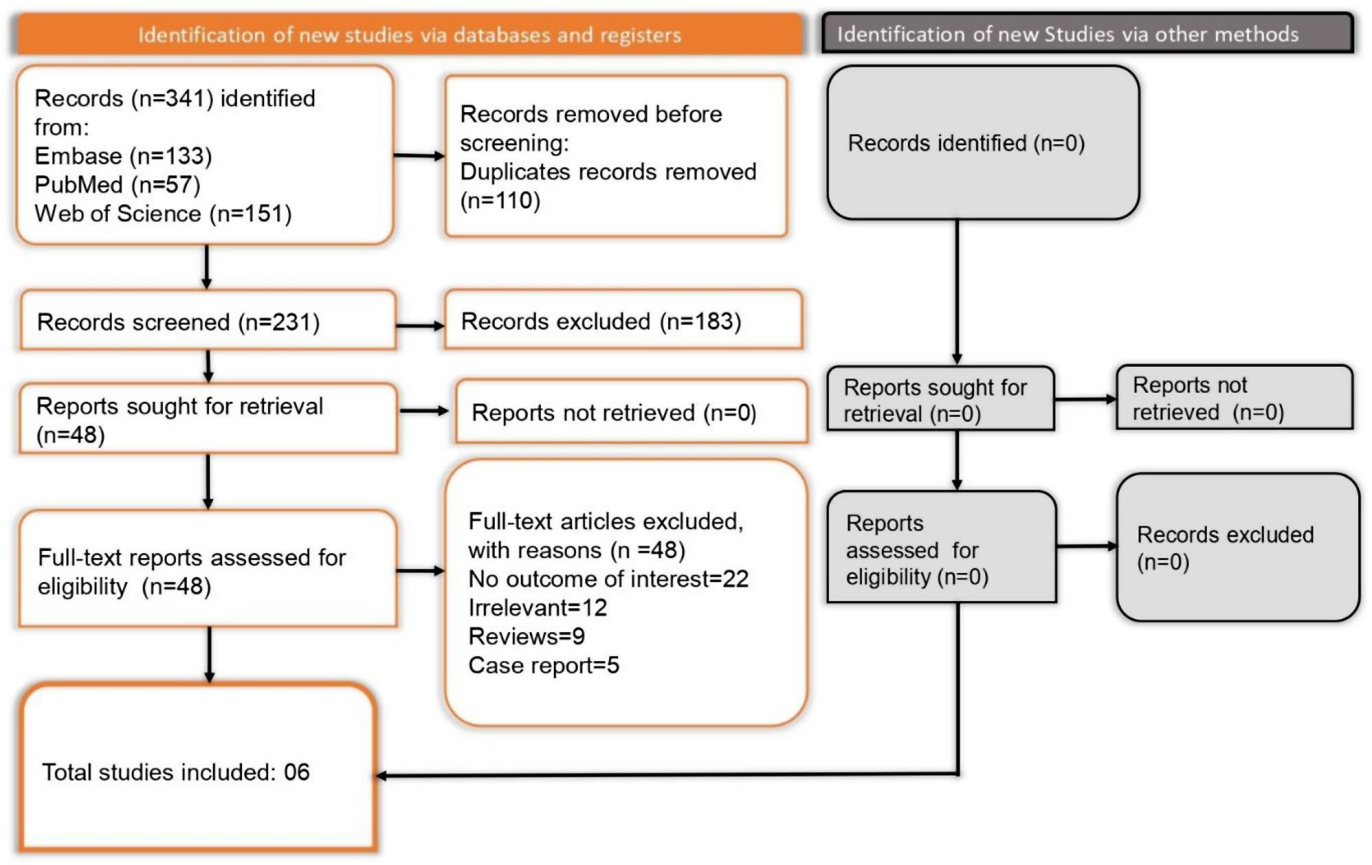
PRISMA flowchart depicting article selection and screening process

A systematic literature search was carried out using three major databases Embase, PubMed, and Web of Science resulting in a total of 341 records (133 from Embase, 57 from PubMed, and 151 from Web of Science). Following the removal of 110 duplicate entries, 231 unique records were screened. Of these, 183 were excluded based on their titles and abstracts as they did not satisfy the inclusion criteria. A total of 48 full-text articles were then reviewed for eligibility, with 22 excluded due to irrelevant outcomes, 12 for lacking relevance to the topic, 9 identified as review articles, and 5 classified as case reports. No additional studies were found through other methods. Ultimately, six studies [14–16, 18–20] met the eligibility criteria and were included in the systematic review, as depicted in the PRISMA flow diagram (Fig. 1).

### Characteristics of included studies

The included studies were conducted in various countries, including the USA and Taiwan, utilizing different observational designs such as cross-sectional studies (*n* = 5) and one retrospective cohort study. Sample sizes varied significantly, ranging from 5,537 to 283,666 participants, drawn from diverse populations, including general adults and participants in large-scale health surveys like NHANES and longitudinal databases such as LHID. Hydrocarbon exposure was assessed using advanced techniques, such as gas chromatography combined with mass spectrometry, to measure compounds like PAHs and volatile organic compounds such as benzene, toluene, ethylbenzene, and xylene (BTEX). Frequently analyzed PAH metabolites included 1-hydroxynaphthalene, 2-hydroxyfluorene, and 1-hydroxypyrene. Associations between hydrocarbon exposure and stroke outcomes were quantified with significant ORs and HRs, alongside CIs. Adjustments for key confounding variables such as age, gender, body mass index (BMI), smoking, physical activity, and serum cotinine levels were routinely incorporated. These results highlight a consistent and strong link between hydrocarbon exposure and increased stroke risk, as summarized in Tables 1 and 2.

**Table 1:** Characteristics of included studies

Study	Country	Study design	Age	Population characteristics	Sample size	Exposure	Key findings
He 2024 [18]	USA	Cross sectional	NA	Participants from NHANES (1999–2018)	17,007	Total BTEX, Benzene, Toluene, Ethylbenzene, Xylene,	Higher BTEX levels in then blood are linked to increased stroke risk, inflammation, dyslipidemia, and lifestyle factors.
Mallah 2022 [14]	USA	Cross sectional	20 years or above	Participants from NHANES (2003–2016)	13,792	Total PAH, 1-Hydroxynaphthalene, 2-Hydroxynaphthalene, 3-Hydroxyfluorene, 2-Hydroxyfluorene, 1-Hydroxypyrene, 1-Hydroxyphenanthrene	Urinary PAH metabolites were linked to cardiovascular diseases like stroke, requiring further study.
Rahman 2023 [15]	USA	Cross sectional	20 years or above	Participants from NHANES (2011–2016)	5,537	1-hydroxynaphthalene 2-hydroxynaphthalene 3-hydroxyfluorene 1-hydroxypyrene	Urinary PAHs and manganese levels were positively linked to higher stroke odds
Shiue 2015 [16]	USA	Cross sectional	20 years or above	Participants from NHANES (2011–2012)	5,560	2-Hydroxyfluorene, 3-Hydroxyfluorene, 9-Hydroxyfluorene, 1-Hydroxyphenanthrene, 2-Hydroxyphenanthrene, 3-Hydroxyphenanthrene, 1-Hydroxypyrene, 1-Hydroxynaphthalene, 4-Hydroxyphenanthrene, 2-Hydroxynaphthalene	Urinary PAHs were linked to stroke, needing further study.
Zhang 2024 [19]	USA	Cross sectional	20 years or above	Participants from NHANES (2007–2016)	7,849	Total PAH, 2-hydroxynaphthalene	High urinary PAH exposure increased diabetes and stroke risk, with diabetes mediating 5% of the stroke risk.
Zhang 2019 [20]	Taiwan	Retrospective cohort	40 years or above	Participants from the LHID (2000)	2,83,666	THC, NMHC	Long-term exposure to THC and NMHC in ambient air was significantly associated with an increased risk of IS.

**Abbreviations**: BTEX - Benzene, Toluene, Ethylbenzene, Xylene; PAH - Polycyclic Aromatic Hydrocarbons; NHANES - National Health and Nutrition Examination Survey; LHID - Longitudinal Health Insurance Database; THC - Total Hydrocarbon; NMHC - Non-Methane Hydrocarbon; IS - Ischemic Stroke

**Table 2:** Exposure, outcome, exposure estimates and covariate adjusted of the included studies

Study	Exposure	Effect size (95% CI for Stroke)	Analysis method	Adjusted factors
He 2024 [18]	Total BTEX,	OR = 0.97, (0.58–1.61)	Gas chromatography coupled with mass spectrometry	Age, gender, race, BMI, education, PIR, smoking, alcohol consumption, physical activity, serum cotinine, diabetes, hypertension, family history of CVD, total energy intake, protein intake, and eGFR
Mallah 2022 [14]	Total PAH	OR = 1.17, (1.02–1.33)	Gas chromatography and high-resolution mass spectrometry	Age (years), gender (male, female), race, education level, marital status, smoking, drinking, BMI, HDL, LDL, total cholesterol (TC), and triglycerides (TG).
	1-Hydroxynaphthalene	OR = 1.89, (1.62–2.20)		
	2-Hydroxynaphthalene	OR = 1.48, (1.28–1.72)		
	3-Hydroxyfluorene	OR = 1.68, (1.44–1.95)		
	2-Hydroxyfluorene	OR = 1.94, (1.66–2.26)		
	1-Hydroxypyrene	OR = 1.41, (1.22–1.64)		
	1-Hydroxyphenanthrene	OR = 1.88, (1.61–2.19)		
Rahman 2023 [15]	1-hydroxynaphthalene 2-hydroxynaphthalene 3-hydroxyfluorene 1-hydroxypyrene	OR = 2.327, (0.961–5.632) OR = 2.449, (1.067–5.622) OR = 2.201, (1.115–4.346) OR = 2.066, (1.037–4.114)	NA	NA
Shiue 2015 [16]	2-hydroxyfluorene	OR = 0.96, (0.63–1.47)	Gas chromatography tandem mass spectrometry	Urine creatinine, age, sex, body mass index, family income-to-poverty ratio, education level, serum cotinine, alcohol use, physical activity level, and subsampling weighting
	3-hydroxyfluorene	OR = 1.00, (0.71–1.41)		
	9-hydroxyfluorene	OR = 1.03, (0.63–1.68)		
	1-hydroxyphenanthrene	OR = 0.93, (0.54–1.60)		
	2-hydroxyphenanthrene	OR = 0.81, (0.45–1.47)		
	3-hydroxyphenanthrene	OR = 0.91 (0.57–1.45)		
	1-hydroxypyrene	OR = 1.05, (0.68–1.63)		
	1-hydroxynapthalene	OR = 1.21, (0.95–1.56)		
	4-hydroxyphenanthrene	OR = 0.73, (0.42–1.28)		
	2-hydroxynapthalene	OR = 1.28, (0.85–1.92)		
Zhang 2024 [19]	Total PAH, 2-hydroxynaphthalene	OR = 1.97, (1.11–3.52) OR = 2.23, (1.17–4.25)	NA	Age, gender, smoking status, drinking status, education level, marital status, physical activity, hypertension, low-density lipoprotein cholesterol, and BMI
Zhang 2019 [20]	THC NMHC	HR = 3.64, (3.56–3.72) HR = 2.21, (2.16–2.26)	NA	PM 2.5

**Abbreviations**: OR - Odds Ratio, HR - Hazard Ratio, CI - Confidence Interval, BTEX - Benzene, Toluene, Ethylbenzene, Xylenes, PAH - Polycyclic Aromatic Hydrocarbons, BMI - Body Mass Index, PIR - Poverty Income Ratio, CVD - Cardiovascular Disease, eGFR - Estimated Glomerular Filtration Rate, HDL - High-Density Lipoprotein, LDL - Low-Density Lipoprotein, TC - Total Cholesterol, TG - Triglycerides, PM 2.5 - Particulate Matter 2.5, THC - Tetrahydrocannabinol, NMHC - Non-Methane Hydrocarbons

### Association of hydrocarbons and their metabolites exposure and risk of stroke

#### Total PAH

The association between total polycyclic aromatic hydrocarbons (PAHs) and stroke risk was examined in several studies. Mallah et al. (2022) [14] found a significant positive association, with an OR of 1.17 (95% CI: 1.02–1.33), suggesting that higher exposure to total PAHs may elevate stroke risk. Similarly, Zhang et al. (2024) [19] reported a strong association, with an OR of 1.97 (95% CI: 1.11–3.52), reinforcing the potential role of total PAH exposure as a stroke risk factor. These findings underscore the importance of total PAH exposure as a determinant of vascular health and emphasize the need for further research to understand the underlying mechanisms.

### Hydroxynaphthalene metabolites

#### 1-Hydroxynaphthalene

The association between 1-hydroxynaphthalene and stroke was evaluated in multiple studies. Mallah et al. (2022) [14] reported a significant positive association, with an OR of 1.89 (95% CI: 1.62–2.20). Similarly, Rahman et al. (2023) [15] observed an increased risk of stroke, with an OR of 2.327 (95% CI: 0.961–5.632). Shiue et al. (2015) [16], however, reported no significant association, with an OR of 1.21 (95% CI: 0.95–1.56). These findings highlight variability in the observed associations, possibly due to differences in study populations, methodologies, or exposure levels.

#### 2-Hydroxynaphthalene

The role of 2-hydroxynaphthalene in stroke risk was explored in several studies. Mallah et al. (2022) [14] identified a significant association, reporting an OR of 1.48 (95% CI: 1.28–1.72). Rahman et al. (2023) [15] further supported this finding, reporting an OR of 2.449 (95% CI: 1.067–5.622). Zhang et al. (2024) [19] also demonstrated a strong association between 2-hydroxynaphthalene and stroke, with an OR of 2.23 (95% CI: 1.17–4.25). However, Shiue et al. (2015) [16] found no significant relationship, reporting an OR of 1.28 (95% CI: 0.85–1.92). These findings highlight variability in the observed associations, potentially due to differences in populations, methodologies, or exposure levels, while emphasizing the need for further research to clarify these relationships.

### Hydroxyfluorene metabolites

#### 2-Hydroxyfluorene

The association between 2-hydroxyfluorene and stroke was evaluated in multiple studies. Mallah et al. (2022) [14] reported a significant positive association, with an OR of 1.94 (95% CI: 1.66–2.26). However, Shiue et al. (2015) [16] found no significant relationship, reporting an OR of 0.96 (95% CI: 0.63–1.47). These discrepancies may reflect differences in exposure assessment methods, population characteristics, or confounding factor adjustments.

#### 3-Hydroxyfluorene

The role of 3-hydroxyfluorene in stroke risk was explored in two studies. Mallah et al. (2022) [14] found a significant positive association, with an OR of 1.68 (95% CI: 1.44–1.95). Similarly, Rahman et al. (2023) [15] supported these findings, reporting an OR of 2.201 (95% CI: 1.115–4.346). In contrast, Shiue et al. (2015) [16] observed no significant association, with an OR of 1.00 (95% CI: 0.71–1.41). The variability in results underscores the need for further investigation to better understand the role of 3-hydroxyfluorene.

#### 9-Hydroxyfluorene

Shiue et al. (2015) [16] examined the association between 9-hydroxyfluorene and stroke, reporting no significant relationship, with an OR of 1.03 (95% CI: 0.63–1.68). This limited evidence highlights the need for additional studies to determine the role of this metabolite in stroke risk.

### Hydroxypyrene metabolites

#### 1-Hydroxypyrene

The association between 1-hydroxypyrene and stroke has been evaluated in multiple studies. Mallah et al. (2022) [14] reported a significant positive association, with an OR of 1.41 (95% CI: 1.22–1.64). Rahman et al. (2023) [15] supported this finding, observing an increased risk of stroke with an OR of 2.066 (95% CI: 1.037–4.114). However, Shiue et al. (2015) [16] found no significant association, reporting an OR of 1.05 (95% CI: 0.68–1.63). These mixed results indicate variability in findings, which may arise from differences in population characteristics, exposure levels, or study methodologies.

### Hydroxyphenanthrene metabolites

#### 1-Hydroxyphenanthrene

The association between 1-hydroxyphenanthrene and stroke was evaluated in multiple studies. Mallah et al. (2022) [14] reported a significant positive association, with an OR of 1.88 (95% CI: 1.61–2.19), suggesting a potential link between this metabolite and stroke risk. However, Shiue et al. (2015) [16] found no significant association, reporting an OR of 0.93 (95% CI: 0.54–1.60). These contrasting results indicate variability across studies and highlight the need for further research to confirm this association.

#### 2-Hydroxyphenanthrene

Shiue et al. (2015) [16] investigated 2-hydroxyphenanthrene and found no significant relationship with stroke, reporting an OR of 0.81 (95% CI: 0.45–1.47). Limited evidence is available for this metabolite, suggesting the need for additional studies to better understand its role in stroke risk.

#### 3-Hydroxyphenanthrene

The association between 3-hydroxyphenanthrene and stroke was examined by Shiue et al. (2015) [16], who reported no significant relationship, with an OR of 0.91 (95% CI: 0.57–1.45). The lack of significant findings warrants further investigation to clarify its potential role.

#### 4-Hydroxyphenanthrene

Shiue et al. (2015) [16] also evaluated 4-hydroxyphenanthrene and found no significant association with stroke, reporting an OR of 0.73 (95% CI: 0.42–1.28). These findings suggest that this metabolite may not be strongly associated with stroke, but further research is needed to confirm this conclusion.

### Total BTEX

The association between total benzene, toluene, ethylbenzene, and xylene (BTEX) exposure and stroke risk was evaluated in a study by He et al. (2024) [18]. They reported no significant association, with an OR of 0.97 (95% CI: 0.58–1.61). This finding suggests that total BTEX exposure may not be a significant independent risk factor for stroke in the population studied. However, the limited number of studies on total BTEX and stroke highlights the need for additional research to better understand its potential impact.

### THC (total hydrocarbons)

Zhang et al. (2019) [20] also evaluated THC and found a significant association with stroke, reporting a hazard ratio HR of 3.64 (95% CI: 3.56–3.72). These findings suggest that THC exposure may be strongly associated with an increased risk of stroke, but further research is needed to confirm this conclusion.

### NMHC (non methane hydrocarbons)

Zhang et al. (2019) [20] further assessed NMHC and observed a significant association with stroke, reporting an HR of 2.21 (95% CI: 2.16–2.26). This indicates a potential strong link between NMHC exposure and stroke risk, warranting additional studies to verify these effects.

## Discussion

The systematic review aimed to evaluate the association between hydrocarbon exposure and the risk of stroke, synthesizing findings from observational studies conducted in diverse geographic locations and employing various methodologies. The reviewed studies highlighted both the potential risks of stroke associated with hydrocarbon exposure, specifically PAHs and VOCs, as well as the variability in study outcomes. Despite this variability, a significant proportion of the studies identified a positive association between hydrocarbon exposure and stroke, particularly with certain PAH metabolites.

The results of this review highlight the critical role of PAH metabolites, such as hydroxynaphthalene, hydroxyfluorene, and hydroxypyrene, in increasing stroke risk. Studies like Mallah et al. (2022) [14] and Rahman et al. (2023) [15] have consistently found significant associations between these metabolites and stroke. These findings align with the well-established mechanisms of hydrocarbon toxicity, which involve oxidative stress, inflammation, and endothelial dysfunction [20], all of which contribute to vascular damage and increased stroke risk. However, the association with hydroxynaphthalene and hydroxyfluorene metabolites is consistently strong, while others, such as hydroxyphenanthrene and hydroxypyrene, show more variability in their impact. For example, Shiue et al. (2015) [16] found no significant correlation between 4-hydroxyphenanthrene and stroke, suggesting that the effects of specific PAH metabolites on stroke risk may depend on factors such as exposure levels, individual susceptibility, or genetic factors. This variability highlights the complexity of studying environmental pollutants and the need for a nuanced understanding of how different PAHs contribute to stroke risk.

A critical study by Zhang et al. (2019) [20] explored the association between THC and non- NMHC exposure and stroke risk. Zhang et al. (2019) found a significant link between long-term exposure to these hydrocarbons in ambient air and an increased risk of ischemic stroke (IS), particularly in individuals aged 40 years and above. The findings emphasize the contribution of hydrocarbons to stroke risk, especially in regions with prolonged exposure to these pollutants in the atmosphere. While the study did not focus on specific PAH metabolites, it provides strong evidence supporting the role of VOCs and hydrocarbons in the pathogenesis of stroke. This reinforces the idea that hydrocarbons, particularly NMHC, are significant contributors to vascular damage and should be considered in stroke prevention efforts, particularly in high-exposure urban and industrial areas.

Additionally, Zhang et al. (2024) [19] focused on the impact of total PAHs and 2-hydroxynaphthalene exposure, finding that high urinary PAH levels were linked to an increased risk of stroke and diabetes, with diabetes acting as a mediator for 5% of the stroke risk. This finding highlights the complex relationship between environmental exposure to PAHs and other comorbidities like diabetes, which can exacerbate the risk of stroke. Zhang et al. (2024) [19] adds valuable insights into how chronic exposure to hydrocarbons, particularly in populations with high environmental pollutant levels, can increase stroke risk, reinforcing the need for public health measures that target PAH exposure and its associated health effects.

The findings from this systematic review align with previous research that has highlighted the harmful effects of environmental pollution on vascular health [21, 22]. This review goes a step further by clearly demonstrating how specific hydrocarbon metabolites, like 1-hydroxynaphthalene and 2-hydroxyfluorene, are linked to an increased risk of stroke, especially in high-risk populations such as industrial workers and those living in highly polluted environments. The research underscores the importance of reducing exposure to these harmful chemicals as a preventive measure against stroke and other related vascular diseases, emphasizing the need for targeted public health initiatives and regulatory measures to protect vulnerable groups from the detrimental effects of hydrocarbon exposure.

A notable strength of this systematic review is its detailed analysis of specific hydrocarbon metabolites, such as 1-hydroxynaphthalene and 2-hydroxyfluorene, which provides valuable insights into the relationship between hydrocarbon exposure and stroke risk. By distinguishing the effects of different metabolites, the review offers a more nuanced understanding of their potential health risks. For instance, Mallah et al. (2022) [14] found a strong association between 1-hydroxynaphthalene and stroke risk in populations exposed to high levels of PAHs, particularly in industrial settings, suggesting a significant link between metabolite levels and stroke incidence. Similarly, Zhang et al. (2024) [19] highlighted 2-hydroxyfluorene as a key metabolite associated with increased stroke risk in individuals with prolonged exposure to PAHs, emphasizing the relevance of this marker in monitoring stroke risk in both occupational and environmental contexts. However, it is important to note that the number of studies examining these specific metabolites is limited, which emphasizes the need for further research to strengthen the evidence base. This refined understanding highlights the importance of targeted interventions and the development of effective biomarkers in public health strategies aimed at reducing stroke risk due to hydrocarbon exposure.

This review highlights important implications for public health and policy development. To reduce the cardiovascular impact of hydrocarbon exposure, it is beneficial to consider strengthening industrial regulations, enhancing air quality standards, and promoting occupational safety measures. Public health initiatives that increase awareness about the potential risks of hydrocarbon exposure and encourage protective behaviours such as avoiding areas with high pollution levels and using personal protective equipment can play a key role in minimizing stroke risk. These strategies, along with ongoing efforts to improve environmental conditions, are crucial for protecting vulnerable populations and reducing the health risks associated with hydrocarbon exposure.

This review has several limitations that must be considered. The included studies varied in design, population demographics, and exposure assessment methods, which may impact the consistency and generalizability of the findings. Additionally, the relatively small number of studies available on this topic limits the strength of the conclusions that can be drawn. The predominance of cross-sectional studies further restricts the ability to establish causal relationships between hydrocarbon exposure and stroke. Moreover, the methods used to measure hydrocarbon exposure, such as urinary biomarkers or environmental monitoring, have limitations related to individual metabolism, timing of sample collection, and variability in laboratory techniques. Furthermore, most of the included studies were conducted in the USA and China, which may limit the generalizability of findings to populations in regions with different pollution levels and exposure patterns. A key limitation is that none of the studies differentiated between ischemic stroke subtypes, such as atherothrombotic, cardioembolic, and lacunar infarcts. Since these subtypes have distinct pathophysiological mechanisms, hydrocarbon exposure may influence them differently. For example, lacunar strokes, which result from small vessel disease, may be more susceptible to endothelial dysfunction and oxidative stress caused by hydrocarbon exposure. In contrast, non-lacunar strokes, including atherothrombotic and cardioembolic strokes, involve large vessel disease and embolic mechanisms, which may interact with environmental pollutants in different ways. Prior studies highlight that lacunar strokes have distinct clinical and pathological characteristics compared to other ischemic stroke subtypes [23].

Given the limitations of the current body of evidence and the complexities associated with hydrocarbon exposure and stroke risk, it is crucial that future research focuses on several key areas. First, studies should investigate whether hydrocarbon exposure has distinct effects on ischemic lacunar versus non-lacunar strokes, as these subtypes have different pathophysiological mechanisms. Understanding these differences is critical for refining risk assessments and developing targeted stroke prevention strategies. Additionally, large-scale, longitudinal cohort studies are essential to establish a clearer causal relationship between hydrocarbon exposure and stroke, particularly among high-risk populations, such as industrial workers and individuals residing in highly polluted urban areas. Further research should also prioritize differentiating between ischemic stroke subtypes and identifying which specific hydrocarbon metabolites pose the greatest risk, with particular emphasis on PAHs and VOCs, which have demonstrated strong associations with stroke in observational studies. Furthermore, mechanistic studies exploring the biological pathways linking hydrocarbon exposure to vascular dysfunction such as oxidative stress, inflammation, and endothelial dysfunction—are necessary to provide critical insights into stroke pathophysiology. Finally, examining gene-environment interactions will help determine whether genetic susceptibility modifies the relationship between hydrocarbons and stroke risk. Addressing these gaps will be pivotal in developing more targeted preventive strategies and informing regulatory policies aimed at reducing exposure to harmful environmental pollutants.

## Conclusion

The evidence on the connection between hydrocarbon exposure and stroke risk is inconsistent, with some studies finding significant associations and others showing no meaningful links. These discrepancies may be due to variations in hydrocarbon types, exposure levels, and study methodologies. Future research should focus on the distinct effects of hydrocarbon exposure on ischemic lacunar versus non-lacunar strokes, and investigate the role of specific metabolites, such as PAHs and VOCs. Large-scale, longitudinal studies are needed to establish causality, particularly in high-risk populations. Additionally, exploring biological mechanisms like oxidative stress and endothelial dysfunction, as well as gene-environment interactions, will be critical. Addressing these gaps is essential for refining preventive strategies and informing policy aimed at reducing harmful exposures.

## Electronic supplementary material

Below is the link to the electronic supplementary material.

Supplementary Material 1

## Data Availability

The data is with the authors and available on request.
